# Spontaneous Slowing and Regressing of Tumor Growth in Childhood/Adolescent Papillary Thyroid Carcinomas Suggested by the Postoperative Thyroglobulin-Doubling Time

**DOI:** 10.1155/2018/6470251

**Published:** 2018-05-16

**Authors:** Toshihiko Kasahara, Akira Miyauchi, Takumi Kudo, Eijun Nishihara, Mitsuru Ito, Yasuhiro Ito, Minoru Kihara, Akihiro Miya

**Affiliations:** ^1^Department of Internal Medicine, Kuma Hospital, 8-2-35 Shimoyamate-dori, Chuo-ku, Kobe 650-0011, Japan; ^2^Department of Surgery, Kuma Hospital, 8-2-35 Shimoyamate-dori, Chuo-ku, Kobe 650-0011, Japan

## Abstract

**Background:**

Children and adolescents with papillary thyroid carcinomas (PTCs) have generally excellent prognoses despite their frequent extended disease. The tumor growth of young patients' PTCs might show spontaneous slowing postoperatively. We compared young PTC patients' postoperative thyroglobulin-doubling time (Tg-DT) with their preoperative hypothetical tumor volume-doubling time (hTV-DT).

**Methods:**

Fourteen PTC patients aged ≤18 years who underwent total thyroidectomy at Kuma Hospital in 1998–2016 had biochemically persistent disease postoperatively. We calculated their Tg-DTs and estimated their preoperative TV-DTs with the tumor size and the patient's age at surgery, presuming that a single cancer cell was present at the patient's birth.

**Results:**

Twelve patients had positive Tg-DTs ranging from 2.0 to 147 years, and the remaining two had negative Tg-DTs, indicating slow growth or even regression. The hTV-DTs were 0.3–0.6 years (median 0.5 years), which were significantly shorter than the Tg-DTs (*p* < 0.001), indicating much faster growth preoperatively. The analyses of the nine patients without radioactive iodine administration (RAI) gave similar results (*p* < 0.01).

**Conclusions:**

Irrespective of RAI, the patients' postoperative Tg-DTs were significantly longer than their preoperative hTV-DTs and were negative values in two patients, indicating that the growth of these young patients' PTCs had spontaneously slowed or even regressed postoperatively.

## 1. Introduction

Children and adolescents with papillary thyroid carcinoma (PTC) have generally excellent prognoses despite their often extended disease status [1–3]. Even when they have distant metastasis, young PTC patients survive for a long time. The excellent prognoses of these patients may be due to their high sensitivity to radioactive iodine (RAI) treatment. In Japan, RAI treatment for childhood and adolescent PTCs has been performed only for patients with distant metastasis, and thyroid ablation with RAI is rarely performed for patients in this age range.

The recurrence of PTC in regional lymph nodes is rather frequent. These recurrences are usually treated surgically without additional RAI treatment. However, even in patients without RAI treatment, the prognoses are good. Papac reported that there was a possibility of spontaneous regression in some tumors such as kidney cancer, malignant melanoma, lymphoma, and leukemia [4]. It is also well known that there is a tendency for spontaneous regression in some pediatric neuroblastomas [5–7].

Collins et al. studied the changes in the tumor sizes of pulmonary metastases over time, and in 1956 they proposed the concept that human tumors grow exponentially [8]. A tumor's growth rate is best expressed as the tumor volume-doubling time (TV-DT). Miyauchi et al. found that the changes in serum calcitonin levels in patients with medullary thyroid carcinoma who had persistent hypercalcitoninemia postoperatively were exponential, which is consistent with Collins' concept, and they reported that the calcitonin-doubling time was a strong prognostic factor [9]. Other research groups confirmed the exponential changes in serum calcitonin and carcinoembryonic antigen (CEA) levels and the prognostic values of the calcitonin-doubling time and the CEA-doubling time [10].

Miyauchi et al. also demonstrated that the serum thyroglobulin (Tg) values measured at a thyrotropin-suppressed condition in PTC patients after total thyroidectomy also changed exponentially over time, and they reported that the Tg-doubling time (Tg-DT) was a strong prognostic factor [11]. Sabra et al. reported that the Tg-DT correlated with the TV-DT in patients with pulmonary metastases of PTC [12]. Tuttle et al. described the tumor volume kinetics of papillary thyroid cancers based on the concept of exponential tumor growth [13]. The TV-DTs of other lesions such as breast cancers, hepatocellular cancers, and prostatic cancers have also been reported [14–16].

We hypothesized that the postoperative tumor growth of PTCs in young patients might slow down spontaneously. We can calculate Tg-DTs, which can be expected to indicate the postoperative tumor growth rate. However, the preoperative growth rates of PTCs in young patients are not known. We estimated the preoperative TV-DT by using the tumor size and the patient's age at surgery, presuming that a single 10-*μ*m dia. cancer cell was present at the patient's birth and that the tumor grew at a constant rate. We call this value the “hypothetical tumor volume-doubling time (hTV-DT).” The actual origin of the cancer would be later than the patient's birth; therefore, the growth before the patient's surgery would have been rapider than this value.

To test our hypothesis, we compared the postoperative Tg-DT with the preoperative hTV-DT in young PTC patients with biochemically persistent disease.

## 2. Materials and Methods

### 2.1. Patients

Between January 1998 and May 2016, 78 PTC patients aged ≤18 years underwent total thyroidectomy at Kuma Hospital. Of these patients, 14 patients without detectable structural disease had four or more detectable serum Tg values measured at serum thyrotropin <0.3 mIU/L postoperatively and undetectable Tg-antibody test results.

We calculated the postoperative Tg-DTs in these 14 patients as described [11]. Serum Tg measurements were performed as routine follow-up tests. We excluded Tg data within 1 month postoperatively and 1 year after RAI administration. The median number of Tg measurements was 6.5.

We calculated the preoperative hTV-DT by using the patient's age at surgery, Y (years), the max. dia. of the tumor, and the dia. of a single cancer cell as 10 *μ*m, that is, 0.01 mm. In general, when a tumor of diameter *D*1 with the first tumor volume (i.e., TV1) grows to a tumor of diameter *D*2 with the second tumor volume (i.e., TV2) over a time period *T*, the TV-DT can be calculated as follows: the tumor volume (TV) is calculated as 4/3  ×  *π*  ×  (*D*/2)^3^, where *D* is the diameter of the tumor. Each TV-DT is given as (log⁡2 × *T*)/log⁡(TV2/TV1) [17]. For the calculation of the hTV-DT values in the present study, *T* = Y (years), *D*1 = 0.01 mm, and *D*2 is the max. dia. of the tumor at surgery.

The present study was approved by the Ethical Committee at Kuma Hospital.

### 2.2. Measurements of Thyroglobulin and Thyroglobulin Antibody

Serum thyroglobulin was measured with a radio-immunoassay (Ab-Beads Thyroglobulin kit, Eiken Chemical, Tokyo) until November 2002 and with an electrochemiluminescence immunoassay (Elecsys Tg kit, Roche Diagnostics, Mannheim, Germany) since November 2002. For the Tg-DT calculation, only Tg values measured with either of these assay methods were used.

Tg-antibody was tested with a radio-immunoassay (Thyroglobulin (Tg) Autoantibody RIA kit, RSR, Pentwyn Cardiff, UK) until March 2008 and with an electrochemiluminescence immunoassay (Elecsys Anti-Tg kit, Roche Diagnostics) since April 2008. Patients with a detectable test result of either of these tests were excluded from the present study.

### 2.3. Statistical Analysis

Two patients had negative Tg-DT values, as described in the Results. This caused a discontinuity problem among the patients with positive and those with negative Tg-DT values. In order to resolve the discontinuity problem, we performed statistical analyses on DT with the reciprocal of DT (i.e., 1/DT), as Barbet et al. described [10]. Differences in 1/DT were calculated using the Wilcoxon signed-rank test. Categorical variables were compared using the Kruskal-Wallis test. These analyses were performed with StatFlex version 6.0 software. All statistical tests were two-sided, with the level of significance set at *p* value < 0.05.

## 3. Results

There were 12 girls and two boys aged 7–18 years with a median of 16.5 years (Table 1). Their tumor sizes ranged from 13 to 46 mm (median 24 mm). All 14 patients underwent central compartment dissection, and 11 patients underwent unilateral (seven patients) or bilateral (four patients) modified neck dissection as well.

All but two of the 14 patients had pathological node metastasis. Only two patients received RAI ablation, 30 mCi and 100 mCi, respectively. Three other patients underwent whole body scintigraphy with a small dose of RAI. One of these five patients showed an accumulation to the lymph node recurrence. The remaining four patients showed no abnormal uptakes.

Five patients developed lymph node recurrence, which were treated surgically. The postoperative follow-up period ranged from 3.5 to 15.8 years (median 8.4 years). None of the patients died of the disease.

**Figure 1:**
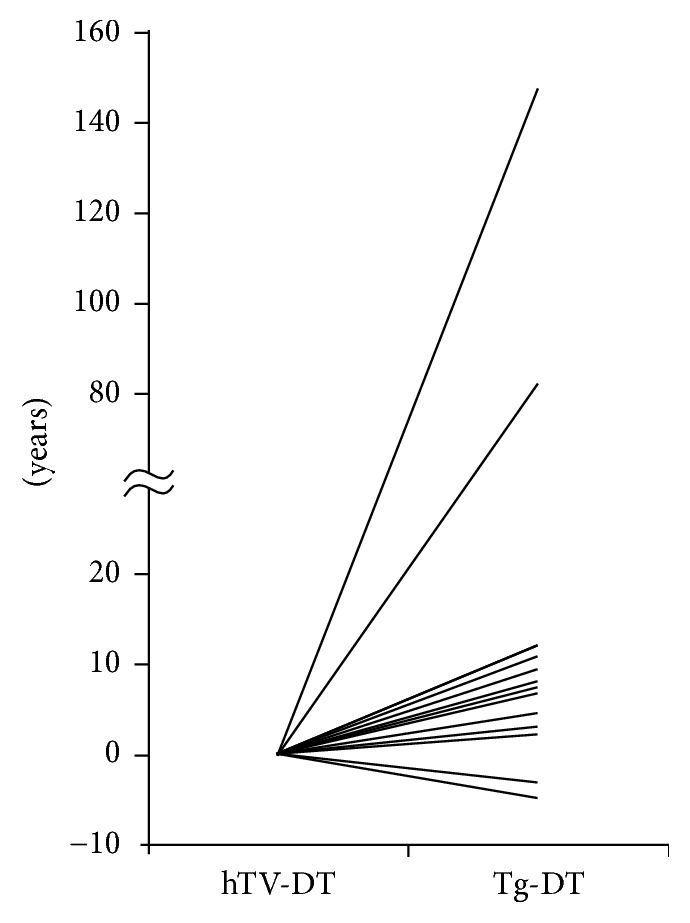
Comparison of hTV-DT and Tg-DT values. hTV-DT: the preoperative hypothetical tumor volume-doubling time; Tg-DT: the postoperative thyroglobulin-doubling time. Two patients showed a decrease in their serum Tg values over time, and thus their Tg-DTs are shown as negative values.

The Tg-DT values were positive values in 12 patients, ranging from 2.0 to 147 years, and two patients showed a decrease in their serum Tg values over time, giving negative Tg-DT values ranging from −3.4 to −4.5 years (Figure 1). The hTV-DTs in the 14 patients ranged from 0.3 to 0.6 years with a median of 0.5 years, suggesting rapid tumor growth before surgery (Figure 1).

**Figure 2:**
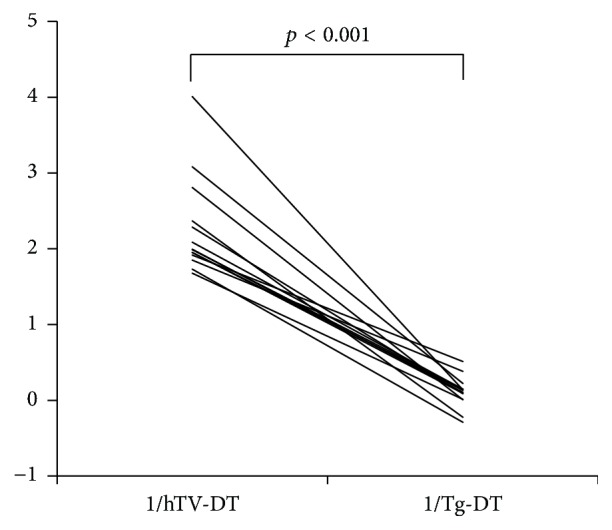
Comparison of the reciprocal of hTV-DT and the reciprocal of Tg-DT. hTV-DT: the preoperative hypothetical tumor volume-doubling time; Tg-DT: the postoperative thyroglobulin-doubling time. The 1/Tg-DT values were significantly smaller than the 1/hTV-DT values in all patients (*p* < 0.001), suggesting a postoperative decrease in tumor growth or even regression.

The 1/Tg-DT values (median 0.12, range −0.29–0.51) were significantly smaller than the 1/hTV-DT values (median 1.99, range 1.68–4.01) (*p* < 0.001, Figure 2), indicating a decrease in tumor growth rate in the 12 patients with positive Tg-DT values and spontaneous regression in the two patients with negative Tg-DT values.

**Figure 3:**
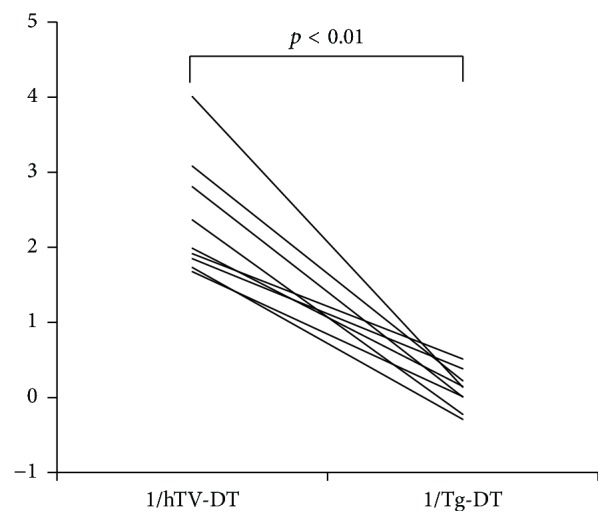
Comparison of the reciprocal of hTV-DT and the reciprocal of Tg-DT in the patients without RAI. hTV-DT: the preoperative hypothetical tumor volume-doubling time; Tg-DT: the postoperative thyroglobulin-doubling time. The 1/Tg-DT values were significantly smaller than the 1/hTV-DT values in all patients (*p* < 0.01), suggesting a postoperative decrease in tumor growth or even regression in patients without administration of RAI.

The analyses of the nine patients without any dose of RAI administration gave similar results (Table 2, Figure 3). In this subset of patients without RAI administration, the 1/Tg-DT values (median 0.14, range −0.29–0.51) were significantly smaller than the 1/hTV-DT values (median 2, range 1.68–4.01) (*p* < 0.01). Two of these patients had negative Tg-DT values, indicating possible tumor regression. These findings indicate spontaneous slowing and even regression of tumor growth in these patients without RAI administration.

## 4. Discussion

Childhood and adolescent PTCs are a mysterious type of cancer. Mazzaferri and Kloos reported that PTC patients aged ≤19 years had high incidences of local and distant recurrences, although their mortality from thyroid cancer was low, and PTC patients aged ≥60 years had both high recurrence rates and high mortality from thyroid cancer [2]. The latter phenomenon in the elderly sounds natural, but the former phenomenon in the youth is confusing. It is well known that young patients with PTC tend to have large tumors, frequent nodal metastasis, and even pulmonary metastasis. However, mortality from thyroid cancer is surprisingly and disproportionally low in young patients despite an advanced disease status.

The most likely explanation for this phenomenon might be that PTCs in young patients are very sensitive to RAI treatment. However, in Japan, the use of thyroid ablation with RAI after thyroidectomy is not common. In the present 14 patients, only two (14%) received thyroid ablation; three received whole body scintigraphy with a small dose of RAI, and nine patients received no RAI at all.

We can express the growth rates of cancers with serum tumor marker-doubling times or with TV-DTs that are calculated with tumor sizes on serial measurements of structural diseases such as pulmonary metastases. These values can usually be obtained only postoperatively. There is generally no direct method to evaluate preoperative tumor growth rates. In the present study, we estimated the preoperative TV-DT using the tumor size and the patient's age at surgery, presuming that a single 10-*μ*m dia. cancer cell was present at the patient's birth. The actual time of the origin of the tumor would be after the patient's birth. Thus, the actual preoperative TV-DT should be smaller, or the actual preoperative growth should be more rapid. One might argue that the growth of a tumor may not have been constant. If there were slow growth periods, there should have been rapid growth periods to grow to the tumor size at surgery. This possibility does not contradict the present contention that the growth of PTCs of young patients spontaneously slows down postoperatively.

In this paper, we describe that 12 young patients with PTC had rather long Tg-DTs and the remaining two had negative Tg-DT values, all of which were significantly longer than the hTV-DTs. This was the case for the patients who were not given any dose of RAI. The hTV-DTs in the present patients ranged from 0.3 to 0.6 years (median 0.5 years). The basal cohort of the present study included 78 PTC patients. The hTV-DTs in these 78 patients ranged from 0.2 to 0.6 years (median 0.5 years; data not shown in detail). These estimates suggest that the PTCs in these young patients had grown very rapidly preoperatively.

One might argue that the serum Tg detected in the present 14 patients came from the residual normal thyroid tissue and not from persistent disease. In our previous study on the Tg-DTs of 426 patients with advanced PTC, 16.2% of the patients showed a decrease in serum Tg over time, resulting in negative Tg-DTs [18]. To address those findings, we studied serum Tg values in 27 consecutive patients with medullary thyroid carcinoma who underwent total thyroidectomy. The postoperative serum Tg level was <0.5 ng/ml in 22 patients (excluding the five patients with positive Tg-antibody) [19]. This suggests that the serum Tg detected in the patients who underwent total thyroidectomy at Kuma Hospital was most unlikely from the residual normal thyroid tissue. Interestingly, the proportion of patients with a negative Tg-DT decreased with age: 20.2% in the patients aged <40 years, 18.4% in the patients aged 40–60 years, and 11.4% in the patients aged ≥60 years [20]. These data also indicate that a postoperative decrease in serum Tg is rather common in young PTC patients.

Pediatric neuroblastoma (stage 4S) is known as a tumor with spontaneous regression. Several groups reported that pediatric astrocytomas also regressed spontaneously [21–24]. Spontaneous tumor growth slowing and even tumor regression in childhood or adolescent patients with PTC might be rather common phenomena.

There are several limitations in this study. The study design was retrospective, and the number of patients was small at 14. However, these patients were recruited from the 78 patients who underwent total thyroidectomy for PTC during an 18-year period at a high-volume hospital for thyroid diseases. Although we determined the Tg-DTs in the 14 patients, none of these patients had structural disease. Therefore, the TV-DTs in these patients were not available. In order to look into the tumor growth before surgery, we propose that the hTV-DT be used. The results of our analyses indicate that the preoperative tumor growth rate was greater than the observed Tg-DT in these patients. However, this finding should be tested in future studies.

## 5. Conclusion

The Tg-DTs in the present 14 PTC patients aged ≤18 years were significantly and definitely longer than their hTV-DTs, irrespective of the use of RAI. Two of the patients showed a decrease in serum Tg values over time without the use of RAI. The present data suggests that the growth of the PTCs in these children and adolescents spontaneously slowed down or even regressed postoperatively.

## Figures and Tables

**Table 1:** Clinical and biochemical features of the 14 childhood or adolescent patients with papillary thyroid carcinomas.

Clinical and biochemical features	Values
Age, yrs	16.5 (7–18)
Tumor size, mm	24 (13–46)
pN0/N1a/N1b	2/1/11
Extrathyroid extension: no/minimal/massive	5/8/1
Thyroid ablation (≥30 mCi)	2 cases
Whole body scintigraphy (≤13 mCi)	3 cases
Follow-up period, yrs	8.4 (3.5–15.8)
Lymph node recurrence	5 cases
Number of Tg measurements	6.5 (4–12)
Tg-DT, yrs	8.3 (−4.5–147)
1/Tg-DT	0.12 (−0.29–0.51)
hTV-DT, yrs	0.5 (0.3–0.6)
1/hTV-DT	1.99 (1.68–4.01)

Values are median (ranges) and numbers of cases, Tg: thyroglobulin, Tg-DT: thyroglobulin-doubling time, and hTV-DT: hypothetical tumor volume-doubling time. Note that 1/Tg-DT was significantly smaller than 1/hTV-DT (*p* < 0.001).

**Table 2:** Comparison of clinical and biochemical features among the patients with and without RAI administration.

Group	A	B	B-1	B-2
	RAI not administered	RAI administered	≤13 mCi	≥30 mCi
	(*n* = 9)	(*n* = 5)	(*n* = 3)	(*n* = 2)
Age, yrs	17 (7–18)	16 (14–18)	16 (14–17)	17 (16–18)
Males/females	1/8	1/4	0/3	1/1
Tumor size, mm	23 (13–46)	25 (15–45)	25 (18–45)	30 (15–45)
Total dose of RAI, mCi	0	13 (6–113)	13 (6–13)	78 (43–113)
Number of Tg measurements	6.5 (4–12)	6 (4–7)	4 (4–7)	6.5 (6-7)
Follow-up period, yrs	8.0 (4.4–13.3)	12.2 (3.5–15.8)	12.2 (3.5–15.8)	11.1 (8.7–13.4)
Tg-DT, yrs	7.1 (−4.5–147.4)	9.1 (6.9–11.5)	9.1 (6.9–11.5)	9.0 (7.5–11)
1/Tg-DT	0.12 (−0.29–0.51)	0.14 (−0.29–0.51)	0.11 (0.087–0.15)	0.11 (0.091–0.13)
hTV-DT, yrs	0.5 (0.25–0.6)	0.5 (0.44–0.51)	0.48 (0.44–0.5)	0.51 (0.51)
1/hTV-DT	1.99 (1.68–4.01)	2 (1.68–4.01)	2.09 (1.99–2.29)	1.95 (1.95)

RAI: radioactive iodine. Values are median (ranges) and numbers of cases. Tg: thyroglobulin, Tg-DT: thyroglobulin-doubling time, and hTV-DT: hypothetical tumor volume-doubling time. Five of the 14 patients underwent RAI administration: ≤13 mCi in 3 patients (B-1) and ≥30 mCi in 2 patients (B-2). Note that 1/Tg-DT was significantly smaller than 1/hTV-DT even in group A (*p* < 0.01). Both the 1/Tg-DTs and 1/hTV-DTs were not significantly different in each group.

## Additional Points

One might think that calculations of DTs are not easy. To solve the problems encountered in the calculation of Tg-DTs and TV-DTs, we created the “Doubling Time and Progression Calculator.” This can be downloaded at Kuma Hospital's website: http://www.kuma-h.or.jp/english/.

## References

[1] Ito Y., Kihara M., Takamura Y. (2012). Prognosis and prognostic factors of papillary thyroid carcinoma in patients under 20 years.

[2] Mazzaferri E. L., Kloos R. T. (2001). Current approaches to primary therapy for papillary and follicular thyroid cancer.

[3] Sung T. Y., Jeon M. J., Lee Y. H. (2017). Initial and dynamic risk stratification of pediatric patients with differentiated thyroid cancer.

[4] Papac R. J. (1998). Spontaneous regression of cancer: possible mechanisms.

[5] Pritchard J., Hickman J. A. (1994). Why does stage 4s neuroblastoma regress spontaneously?.

[6] Okazaki T., Kohno S., Mimaya J.-I. (2004). Neuroblastoma detected by mass screening: The Tumor Board's role in its treatment.

[7] Haas D., Ablin A. R., Miller C., Zoger S., Matthay K. K. (1988). Complete pathologic maturation and regression of stage ivs neuroblastoma without treatment.

[8] Collins V. P., Loeffler R. K., Tivey H. (1956). Observations on growth rates of human tumors.

[9] Miyauchi A., Onishi T., Morimoto S. (1984). Relation of doubling time of plasma calcitonin levels to prognosis and recurrence of medullary thyroid carcinoma.

[10] Barbet J., Campion L., Kraeber-Bodéré F., Chatal J.-F. (2005). Prognostic impact of serum calcitonin and carcinoembryonic antigen doubling-times in patients with medullary thyroid carcinoma.

[11] Miyauchi A., Kudo T., Miya A. (2011). Prognostic impact of serum thyroglobulin doubling-time under thyrotropin suppression in patients with papillary thyroid carcinoma who underwent total thyroidectomy.

[12] Sabra M. M., Sherman E. J., Tuttle R. M. (2017). Tumor volume doubling time of pulmonary metastases predicts overall survival and can guide the initiation of multikinase inhibitor therapy in patients with metastatic, follicular cell-derived thyroid carcinoma.

[13] Tuttle R. M., Fagin J. A., Minkowitz G. (2017). Natural history and tumor volume kinetics of papillary thyroid cancers during active surveillance.

[14] Zhang S., Ding Y., Zhu Q., Wang C., Wu P., Dong J. (2017). Correlation factors analysis of breast cancer tumor volume doubling time measured by 3D-ultrasound.

[15] An C., Choi Y. A., Choi D. (2015). Growth rate of early-stage hepatocellular carcinoma in patients with chronic liver disease.

[16] Schmid H., McNeal J. E., Stamey T. A. (1993). Observations on the doubling time of prostate cancer. The use of serial prostate‐specific antigen in patients with untreated disease as a measure of increasing cancer volume.

[17] Schwartz M. (1961). A biomathematical approach to clinical tumor growth.

[18] Miyauchi A., Kudo T., Kihara M. (2013). Relationship of biochemically persistent disease and thyroglobulin-doubling time to age at surgery in patients with papillary thyroid carcinoma.

[19] Tomoda C., Miyauchi A. (2012). Undetectable serum thyroglobulin levels in patients with medullary thyroid carcinoma after total thyroidectomy without radioiodine ablation.

[20] Miyauchi A., Kudo T., Hirokawa M. (2013). Ki-67 labeling index is a predictor of postoperative persistent disease and cancer growth and a prognostic indicator in papillary thyroid carcinoma.

[21] Samadian M., Bakhtevari M. H., Haddadian K., Alavi H. A., Rezaei O. (2016). Spontaneous complete regression of hypothalamic pilocytic astrocytoma after partial resection in a child, complicated with Stevens-Johnson syndrome: a case report and literature review.

[22] Loh J.-K., Lieu A.-S., Chai C.-Y. (2013). Arrested growth and spontaneous tumor regression of partially resected low-grade cerebellar astrocytomas in children.

[23] Foroughi M., Hendson G., Sargent M. A., Steinbok P. (2011). Spontaneous regression of septum pellucidum/forniceal pilocytic astrocytomas—possible role of Cannabis inhalation.

[24] Palma L., Celli P., Mariottini A., Chumas P. D. (2004). Long-term follow-up of childhood cerebellar astrocytomas after incomplete resection with particular reference to arrested growth or spontaneous tumour regression.

